# The Dermatological Effects and Occupational Impacts of Personal Protective Equipment on a Large Sample of Healthcare Workers During the COVID-19 Pandemic

**DOI:** 10.3389/fpubh.2021.815415

**Published:** 2022-01-24

**Authors:** Paolo Emilio Santoro, Ivan Borrelli, Maria Rosaria Gualano, Ilaria Proietti, Nevena Skroza, Maria Francesca Rossi, Carlotta Amantea, Alessandra Daniele, Walter Ricciardi, Concetta Potenza, Umberto Moscato

**Affiliations:** ^1^Department of Woman and Child Health and Public Health, Fondazione Policlinico Universitario A. Gemelli Istituto di Ricovero e Cura a Carattere Scientifico, Rome, Italy; ^2^Department of Life Sciences and Public Health, Section of Occupational Health, Università Cattolica del Sacro Cuore, Rome, Italy; ^3^Department of Life Sciences and Public Health, Università Cattolica del Sacro Cuore, Rome, Italy; ^4^Department of Public Health Sciences and Paediatrics, University of Torino, Torino, Italy; ^5^Dermatology Unit “Daniele Innocenzi”, “A. Fiorini” Hospital, Terracina, Italy; ^6^Department of Life Sciences and Public Health, Section of Hygiene, Università Cattolica del Sacro Cuore, Rome, Italy

**Keywords:** occupational health, personal protective equipment, COVID-19, skin reactions, occupational dermatology

## Abstract

**Introduction:**

Working during the Sars-CoV-2 pandemic healthcare workers (HCWs) had to wear Personal Protective Equipment (PPEs) for extended periods of time, leading to an increase in dermatological reactions. The study evaluates the prevalence of adverse skin reactions to PPEs among Italian healthcare workers during the COVID-19 pandemic, and aims to determine whether prolonged PPEs usage poses a significant occupational health risk, by measuring the loss of work days and the eligibility of workers that requested health surveillance due to dermatological PPEs reactions.

**Materials and Methods:**

An online *ad hoc* questionnaire was administered to a sample of Italian HCWs. Questions verted on sociodemographic characteristics, PPEs usage, and occupational well-being. Descriptive analyses and logistic regressions were performed to explore possible associations between variables.

**Results:**

Two types of PPEs, Gloves and Masks, were tested. The sample included 1,223 interviewed HCWs, 1,184 gave their consent for participation. A total of 90 medical surveillance visits were requested due to PPEs related dermatological issues: in 30 cases were recognized limitations in working duties and in one case the worker was deemed not fit to keep working. Furthermore, 25 workers had a loss of occupational days due to dermatological issues. A statistically significant correlation was observed with being a nurse or midwife (OR = 1.91, IC = 1.38–2.63, *p* < 0.001), and being female (OR = 2.04, IC = 1.49–2.78, *p* < 0.001), which acted as risk factors.

**Discussion:**

The enhanced protection measures put in place during the COVID-19 pandemic, highlight the importance of occupational dermatology. This study could contribute to assess the issue, aiming to develop better prevention strategies in the workplace in order to improve well-being of HCWs and reduce the impact of dermatological adverse reactions to PPEs.

## Introduction

Coronavirus disease 2019 (COVID-19) originated in Wuhan, China in December 2019. Within a short amount of time, hundreds of thousands of cases were diagnosed around the world, causing the World Health Organization to announce it as an infectious disease pandemic on January 30, 2020.

The main form of human-to-human transmission occurs through respiratory droplets expelled by an infected individual; hence, coughing and sneezing render SARS-CoV-2 airborne, putting non-infected individuals at risk of contracting the disease (1–3). Additionally, data have indicated that SARS-CoV-2 transmission can also occur as a result of contact with contaminated inanimate objects, also known as fomite transmission (4, 5).

The most important strategy to undertake the risk of contagion is frequent handwashing, using portable hand sanitizer, practicing respiratory hygiene (i.e., covering their cough), avoiding crowds and extended use of personal protective equipment (PPE), such as masks, gloves, goggles, face shields, and bonnets.

Due to the uncertainty of the infection status of patients or the direct contact with COVID-19 patients (6), healthcare workers (HCWs), are mandated to wear PPE to markedly reduce the infection risk (7–9).

Many healthcare workers, aside from a major risk of contracting the disease, reported added stress from adverse effects of prolonged PPE usage, such as headaches, breathing difficulty and impaired cognition. It also interferes with vision, communication and thermal equilibrium (10–12).

These enhanced protection measures during COVID-19 emergency highlight the importance of occupational dermatology (13). It has been reported that up to 97% of HCWs showed skin lesions (14), including acne, skin breakdown, rashes, contact and pressure urticaria, rosacea, perioral dermatitis, contact dermatitis, or aggravation of pre-existing skin disorders.

The most commonly affected areas were the hands, cheeks, and nasal bridge (10, 15).

Skins problems often have a significant impact on emergency management, as they effect patients' quality of life and are potentially able to reduce the effective workforce (10–12).

Prolonged usage of PPE can exacerbate or cause acne vulgaris (16). The tight seal and humid environment created by masks, particularly N95s, aggravates acne (also known colloquially as maskne) (17, 18). This is likely because pressure on the skin can rupture comedones and block pilosebaceous ducts. Moreover, the humid microclimate within the mask is ideal for bacterial growth and prevents filaggrin (FLG) breakdown, which contributes to skin barrier disruption (18).

Atopic dermatitis (AD) and irritant contact dermatitis (ICD) are common types of eczema that are characterized by pruritus, eczematous lesions, xerosis, and lichenification. AD is a chronic relapsing inflammatory skin condition that often develops at a young age, while ICD is caused by direct contact of the skin with environmental, chemical, or physical agents that disrupt the epidermal barrier (19, 20). AD and ICD can be exacerbated or caused by wearing PPE for long periods of time (15, 21).

Skin reactions to gloves included complaints of dry skin, itch, and rash (22).

Hand eczema (HE) is the most common form of ICD (23). Anionic surfactants, commonly found in hand soaps, disrupt the stratum corneum by damaging proteins and the processing of new lipids, allowing for greater penetration of irritants and Transepidermal Water Loss (TEWL) (24). Likewise, extended exposure to water disrupts the stratum corneum's lipid structure and increases skin permeability (25). Other irritants, such as organic solvents used in hand sanitizers, strip away lipids from the stratum corneum, although they are less damaging compared to harsh detergents (26).

Wearing gloves or having wet hands for >2 h during work hours, or hand washing 10–20 times daily, is generally accepted to be quantitatively sufficient for triggering of irritant contact dermatitis (27–29). Additionally, the timeframe that an activity can be sustained is decreased when wearing masks and PPE (10, 11, 16, 21).

These adverse effects are mainly caused by the hyperhydration effects of PPE, friction, epidermal barrier breakdown, and contact reactions. All of these can aggravate pre-existing skin diseases or cause new ones, many of which can be controlled with proper moisturization. Moisturizers treat damaged skin by repairing the stratum corneum, increasing hydration, and reducing trans epidermal water loss (TEWL) (24). However, the main obstacle remains poor adherence to skin care recommendations (30–33).

It is acknowledged that PPE items are designed for single use. However, the reality during the course of the pandemic is that reuse has been undertaken by many health care workers across the world out of necessity, who were challenged to rationally use the limited supplies by decontaminating and reprocessing them (34). Improper or inadequate decontamination of equipment before reuse is unsafe and can pose serious threats (35).

The study aims to determine the prevalence of adverse skin reactions to PPE among Italian healthcare workers during the COVID-19 pandemic and characterize them, hence determining whether prolonged PPE use poses a significant occupational health risk; this risk was assessed by measuring the loss of work days and the eligibility of workers that requested health surveillance due to dermatological PPEs reactions. We also intend to identify potential predictors of cutaneous adverse reactions due to the PPE usage, and corrective actions to be applied in order to reduce this occupational risk.

## Materials and Methods

### Sample and Questionnaire

The study is a cross-sectional study; a questionnaire was administered to healthcare workers using an online platform. Participants were recruited by convenience among the workplace circle of the authors and were invited to send the questionnaire to other colleagues, therefore using snowball sample recruitment. The participants were informed about the aim of the study and gave their consent to participate before accessing the survey. A total of 1,184 valid answers were received and thus included in the study.

The questionnaire included 29 questions, divided into four sections.

The first section was made up of seven questions. The first five questions assessed the participant's gender, age, mansion, working sector, and if the healthcare professional assisted Sars-CoV-2 patients; two additional questions investigated if the participant had a history of dermatological illness and, if they had, which one.

The second section investigated the use of gloves during work hours, with seven questions pertaining to: gloves type and usage time, if cutaneous hand reactions were observed and which ones, times hands were washed and times hand gel was used, and use of hand cream.

The third section included questions about the use of face masks, with nine questions investigating: mask type and usage time, reusage of mask and if it was disinfected before reusing it (if it was, which disinfectant was used), if adverse reactions to the mask were observed and which ones, and if face creams (and which type) were used.

The fourth and final section focused on the occupational health aspect, with six questions investigating if the healthcare workers requested medical surveillance, their eligibility, if work days were missed due to adverse reactions to PPEs and how many, if their company had given instructions about the management of adverse reactions to PPEs, and if the creams and/or lotions used had been supplied by their company.

### Statistical Analyses

Skewness and kurtosis were used to investigate the distribution of the collected data and the Saphiro-Wilk test was used to investigate normal distribution. The variables that were normally distributed were: gender, working with Sars-CoV-2 patients, Adverse dermatological effects to PPEs, Recycling mask, Washing recycled mask, Adverse dermatological reaction to the hands, Type of hand cream used, Requesting medical surveillance for and adverse dermatological reaction, Type of reaction for which medical surveillance was requested, and Number of lost work days. All other variables were not-normally distributed.

Descriptive statistics were used to assess participants' socio-demographic data, and frequencies and percentages were defined.

Pearson bivariate correlations were performed to check multi-collinearity and to give some preliminary information into relationships between dermatological disease and the use of PPE; *p*-values were considered significant if they were ≤0.05.

In a second stage the significant predictors of the first stage were entered together in multiple logistic regression models. Data were stratified by gender, age, occupational group and sector, time of PPE usage, type and material of PPE. Crude odds ratios (ORs) and adjusted ORs for all the other entered variables, along with 95% confidence intervals, were calculated. To analyse the collected data, we used the STATA 16 statistical package.

## Results

The sample included 1,223 interviewed HCWs, 1,184 gave their consent for participation (response rate: 96,8%).

Of them, 257 (21.71%) were males and 927 (78.29%) females. The age range was between 21 and 68 years, with a mean of 43.37 (SD 10.94) years. Concerning occupational groups, the healthcare workers were: 332 (28.04%) physicians, 772 (65.20%) nurses/midwives, 80 (6.76%) other professionals. Regarding the working sector, workers were distributed as follows: 367 (31%) were employed in Hospital Wards, 253 (21.37%) in day hospital, 114 (9.63%) in Intensive Care Research, 88 (7.43%) in Emergency Room, 71 (6%) in Surgery, 41 (3.46%) in Delivery Room and 250 (21.11%) in other sectors. Among all the participants, 292 (24.66%) reported a dermatological illness, grouped in four different pathological issues: 45 (15.421%) had Psoriasis, 54 (18.49%) Eczema, 38 (13.01%) Acne, 48 (126.44%) had Seborrheic Dermatitis and 107 (36.64%) other reactions.

Making a distinction based on personal history, 38 (10.67%) participants with no personal history of dermatological reactions had issues related to PPE, and 254 (30.68%) participants with a personal history of dermatological reactions had an adverse reaction to PPE.

From an Occupational Health standpoint, 90 (7.6%) workers requested a health surveillance visit. Among these participants, two workers were deemed unfit to keep working (one of them requested health surveillance due to PPE-related issues, and one did so for other reasons), 30 were given limitations to their daily working activities, and 55 were deemed fit to work. For three workers who requested health surveillance, the eligibility data was not reported.

Out of the 90 participants who requested a health surveillance visit, 56 did so for PPE-related issues; among these, 49 (87.5%) had a personal history of dermatological reactions, in 25 of them a limitation to working activities was established and one of them was deemed not fit to work (Table 1). Having personal history of dermatological problems was associated with requesting a health surveillance visit; the correlation was statistically significant (*p* > 0.001).

**Table 1:** Univariate analysis of frequencies for *Adverse Dermatological Reaction, Medical Surveillance* and *Eligibility*.

		**Adverse dermatological effect (** ***N*** **, %)**		
		**No**	**Yes**	***p*-value**
Medical surveillance	No	349	779	0.003^*^
		30.94	69.06	
	Yes	7	49	
		12.50	87.50	
	Total	356	828	
		30.07	69.93	
Eligibility	Limitations	3	27	0.216
		10.00	90.00	
	Suitable	30	25	
		54.55	45.45	
	Not Suitable	4	1	
		80.00	20.00	
	Total	37	53	
		41.11	58.89	

* *Statistically significant value p ≤ 0.05*.

In 25 (2.11%) workers, a loss of working days due to dermatological issues was observed; 15 participants missed work for <7 days, seven participants missed between 7 and 20 days of work, and three participants missed more than 20 days of work due to dermatological illnesses. A previous dermatological illness was present in 6 (85.71%) workers who missed between 7 and 20 working days; the correlation was statistically significant (*p* = 0.031). A personal history of dermatological disease was also significantly (*p* = 0.038) correlated with mansion, as 209 (27%) nurses reported previous dermatological illnesses (Table 2).

**Table 2:** Univariate analysis of frequencies for *Previous Dermatological Illness, Number of lost days of work* and *Job*.

		**Previous dermatological illness (** ***N*** **, %)**		
		**No**	**Yes**	***p*-value**
Number of lost work days	<7	11	4	0.031^*^
		73.33	26.67	
	7–20	1	6	
		14.29	85.71	
	>20	2	1	
		66.67	33.33	
	Total	14	11	
		56.00	44.00	
Job	Other	64	16	0.038^*^
		80.00	20.00	
	Physician	265	67	
		79.82	20.18	
	Nurse-midwife	563	209	
		72.93	27.07	
	Total	892	292	
		75.34	24.66	

* *Statistically significant value p ≤ 0.05*.

A logistic regression was performed to test correlation between adverse dermatological reactions and the following variables: occupational group, working environment, age and gender. A statistically significant correlation was observed with being a nurse or midwife (OR = 1.91, IC = 1.38–2.63, *p* < 0.001), and being female (OR = 2.04, IC = 1.49–2.78, *p* < 0.001), which acted as risk factors. As protective factors, a statistically significant correlation was observed with working in day hospital (OR = 0.52, IC = 0.29–0.94, *p* = 0.031), being between 31 and 40 years of age (OR = 0.56, IC = 0.36–0.87, *p* = 0.009), or being over 50 years of age (OR = 0.46, IC = 0.30–0.72, *p* = 0.001).

Two types of PPE were tested: Gloves and Masks.

As far as gloves were concerned, 591 (50%) participants reported dermatological reactions on the hands. These reactions were more frequent in participants between 41 and 50 years of age (29.61%), female (83.42%), working with Sars-CoV-2 patients (63.62%). Most participants (42.64%) had a reaction between 3 and 6 h of wearing gloves, most reactions happened in patients who wore nitrile gloves (57.53%), in participants who washed their hands more than 10 times per day (63.79%), or used hydroalcoholic gel more than 10 times per day (61.93%). People using a hand cream had more dermatological reactions (72.30%) than those who didn't use any (Table 3).

**Table 3:** Univariate analysis of frequencies for *Adverse dermatological reactions to the hands* and *Adverse dermatological reactions to the mask*.

***N* = 1,184**		**Adverse dermatological reactions to the hands (** ***N*** **, %)**			**Adverse dermatological reactions to the mask (** ***N*** **, %)**		
		**No**	**Yes**	***p*-value**	**No**	**Yes**	***p*-value**
Age	21–30	82	128	0.003^*^	84	126	0.002^*^
		39.05	60.95		40.00	60.00	
	31–40	137	129		124	142	
		51.50	48.50		46.62	53.38	
	41–50	179	175		150	204	
		50.56	49.44		42.37	57.63	
	51+	195	159		193	161	
		55.08	44.92		54.52	45.48	
Gender	Male	159	98	0.000^*^	175	82	<0.001^*^
		61.87	38.13		68.09	31.91	
	Female	434	493		376	551	
		46.82	53.18		40.56	59.44	
Working with COVID-19 patients	No	338	215	<0.001^*^	309	244	<0.001^*^
		61.12	38.88		55.88	44.12	
	Yes	255	376		242	389	
		40.41	59.59		38.35	61.65	
Previous dermatolo-gical illness	Psoriasis	16	29	0.018^*^	19	26	0.045^*^
		35.56	64.44		42.22	57.78	
	Eczema	9	45		21	33	
		16.67	83.33		38.89	61.11	
	Acne	17	21		5	33	
		44.74	55.26		13.16	86.84	
	Seb-derm	19	29		17	31	
		39.58	60.42		35.42	64.58	
	Other	28	79		33	74	
		26.17	73.83		30.84	69.16	
Time wearing gloves	<1 h	113	38	<0.001^*^			
		74.83	25.17				
	1 < h < 3	157	122				
		56.27	43.73				
	3 < h < 6	210	252				
		45.45	54.55				
	>6	112	179				
		38.49	61.51				
Type of gloves used	Lattex no dust	206	179	0.284			
		53.51	46.49				
	Lattex dust	37	46				
		44.58	55.42				
	Nitrile	331	340				
		49.33	50.67				
	Other	19	25				
		43.18	56.82				
Times hands washed per day	Never	0	1	<0.001^*^			
		0.00	100.00				
	<5	74	40				
		64.91	35.09				
	5 < *t* < 10	213	173				
		55.18	44.82				
	>10	306	377				
		44.80	55.20				
Times hand gel was used per day	<5	72	56	0.066			
		56.25	43.75				
	5 < *t* < 10	192	169				
		53.19	46.81				
	>10	329	366				
		47.34	52.66				
Using Hand Cream	No	272	146	<0.001^*^			
		65.07	34.93				
	Yes	321	445				
		41.91	58.09				
Time wearing mask	<1 h				12	2	<0.001^*^
					85.71	14.29	
	1 < h < 3				21	7	
					75.00	25.00	
	3 < h < 6				143	94	
					60.34	39.66	
	>6				373	530	
					41.31	58.69	
Type of mask	Surgical mask				366	386	0.299
					48.67	51.33	
	FFP2 no valve				162	213	
					43.20	56.80	
	FFP2 valve				8	7	
					53.33	46.67	
	FPP3 no valve				7	14	
					33.33	66.67	
	FFP3 valve				3	7	
					30.00	70.00	
	Other type				4	6	
					40.00	60.00	
Recycling mask	No				209	301	0.001^*^
					40.98	59.02	
	Yes				342	331	
					50.82	49.18	
Washing recycled mask	No				433	523	0.079
					45.29	54.71	
	Yes				118	110	
					51.75	48.25	
Type of gel used on mask	NaClO				32	26	0.822
					55.17	44.83	
	70% alcohol				60	62	
					49.18	50.82	
	Alcohol isopropile				5	5	
					50.00	50.00	
	Alcohol gel				10	14	
					41.67	58.33	
	Other				10	8	
					55.56	44.44	
Using face cream	No				326	221	<0.001^*^
					59.60	40.40	
	Yes				223	411	
					35.17	64.83	
Type of face cream	Soothing				14	142	<0.001^*^
					8.97	91.03	
	Moisturizers			222	268		
					45.31	54.69	

* *Statistically significant value p ≤ 0.05*.

A logistic regression was performed to test the correlation between adverse hand reactions and the following variables: working with Sars-CoV-2 patients, type of previous dermatological illness, time wearing gloves, times hands were washed during a working day, use of hand cream, age, and gender (Table 4). Our results showed that working in contact with Sars-CoV-2 patients was a risk factor for an adverse dermatological reaction to the hands (OR = 2.06, CI = 1.18–3.60, *p* = 0.011), and the correlation was statistically significant. Wearing gloves between 3 and 6 h was a statistically significant risk factor (OR = 3.02, CI = 1.04–8.79, *p* = 0.042) for adverse hand reactions. Out of the 292 patients with a previous dermatological issue, 203 (69.52%) had adverse hand reactions; concerning the pathology subgroups, a statistically significant correlation with hand adverse reactions showed that having a personal history of Acne (OR = 0.24, CI = 0.08–0.66, *p* = 0.006) or Seborrheic Dermatitis (OR = 0.31, CI = 0.12–0.82, *p* = 0.018) was protective for hand reactions.

**Table 4:** Logistic regression analysis for *Adverse dermatological reactions to the hands* and *Adverse dermatological reactions to the mask*.

		**Adverse derm reactions to hands**		**Adverse derm reactions to mask**	
		**OR**	**(95% Conf interval)**	**OR**	**(95% Conf interval)**
Working with COVID-19 patients	No	1	–	1	–
	Yes	2.062	1.18–3.603^*^	2.336	1.048–5.206^*^
Previous dermatological illness	Psoriasis	0.401	0.15–1.07	0.909	0.278–2.971
	Eczema	1	–	1	–
	Acne	0.237	0.085–0.664^*^	6.875	1.132–41.736^*^
	Seb-derm	0.309	0.117–0.819^*^	1.847	0.494–6.913
	Other	0.554	0.232–1.325	1.688	0.559–5.096
Time wearing gloves	<1 h	1	–		
	1 < h < 3	1.628	0.54–4.909		
	3 < h < 6	3.027	1.042–8.795^*^		
	>6	1.823	0.589–5.643		
Times hands washed per day	<5	1	–		
	5 < *t* < 10	1.622	0.601–4.375		
	>10	2.022	0.776–5.269		
Using hand cream	No	1	–		
	Yes	1.476	0.816–2.672		
Time wearing mask	<3 h			1	–
	>3 h			2.674	0.367–19.494
Recycling mask	No			1	–
	Yes			0.919	0.406–2.079
Using face cream	No			1	–
	Yes			3.994	1.004–15.895^*^
Type of face cream	Soothing			1	–
	Moisturizers			0.125	0.041–0.386^*^
Age	21–30	1	–	1	–
	31–40	0.695	0.282–1.716	1.003	0.297–3.388
	41–50	0.874	0.375–2.038	1.005	0.3–3.367
	51+	0.807	0.338–1.926	1.457	0.432–4.916
Gender	Male	1	–	1	–
	Female	0.804	0.391–1.654	3.631	1.127–11.695^*^

* *Statistically significant value p ≤ 0.05*.

We observed adverse reactions to the face mask in 633 (53.46%) participants. This type of adverse reactions were more frequent in workers between 41 and 50 years of age (32.22%), females (87.04%), working with Sars-CoV-2 patients (61.45%), wearing masks for more than 6 h per day (83.73%), using surgical masks (60.98%). Reactions to the face mask were more frequent in participants who recycled the same mask (52.37%), and did not disinfect it for re-usage (82.62%). More adverse reactions were observed in participants using face creams (65.03%), and more specifically moisturizers (65.36%) over soothing creams (Table 3).

Another logistic regression was performed to test the correlation between adverse face mask reactions and the following variables: working with Sars-CoV-2 patients, type of previous dermatological illness, time wearing mask, recycling face masks, use of face cream, type of face cream used, age, and gender (Table 4). Our results showed a statistically significant correlation between adverse dermatological face reactions and the following variables that acted as risk factors: working with Sars-CoV-2 patients (OR = 2.33, IC = 1.04–5.21, *p* = 0.038), having Acne as a previous dermatological illness (OR = 6.87, IC = 1.13–41.73, *P* = 0.036), using a face cream (OR = 3.99, IC = 1.00–15.89, *p* = 0.049), and being female (OR = 3.63, IC = 1.13–11.69, *p* = 0.031). Using a moisturizer (OR = 0.12, IC = 0.04–0.38, *p* < 0.01) was a statistically significant protective factor for adverse reactions to face masks.

## Discussion

Out of the workers who requested health surveillance due to PPE-related reasons, 87.5% had a personal history of dermatological reactions. In 2.11% of healthcare professionals a loss of working days was observed, because a limitation to working activities was established, and one worker was deemed not fit to work. Adverse dermatological reactions to the hands were higher for healthcare professionals who worked in contact with Sars-CoV-2 patients and wore gloves between 3 and 6 h, and lower for workers with a personal history of Acne or Seborrheic Dermatitis. Adverse dermatological reactions to the face were higher for female healthcare professionals, in workers interacting with Sars-CoV-2 patients, in who had Acne, or used a face cream; masks reactions were lower in workers using a moisturizer.

To our knowledge, this is the first study to investigate dermatological reactions caused by PPEs in an occupational health perspective. A personal history of dermatological illness was significantly associated with requesting a health surveillance visit, with missing between 7 and 20 working days and with the profession (being a nurse). Out of the 90 workers who requested health surveillance for PPE-related dermatological issues, 30 were given limitations to their working activities and one was deemed not fit to work. These results highlight the need to establish a better line of action in preventing PPE-related issues in professionals who already have a dermatological illness, as well as the need to promptly intervene when a dermatological issue ensues to prevent the worsening of cutaneous symptoms and illnesses; this statement is underlined by our finding that a loss of working days due to dermatological issues was observed in 25 workers because the occupational physician deemed the healthcare professional unfit or only partially fit for work.

A review from Spagnolo et al. has highlighted the growing importance of medical surveillance and occupational medicine during the Sars-CoV-2 pandemic (36). As suggested in this review, medical surveillance is essential, and it could be a fundamental instrument in early identification of individual susceptibility to PPEs usage and to prevent the onset of adverse reactions or chronic clinical conditions. Furthermore, medical surveillance could be a useful tool to identify those HCWs who need a targeted training in the use of PPEs, specific for their work tasks and routine.

A study conducted in 2020 by di Altobrando et al. has highlighted a growing problem in dermatological reactions to masks; the researchers proposed a series of precautions to avoid skin reactions, including but not limited to: not wearing fabric masks, not reusing masks, not applying disinfectant gel on the masks' surface, use emollient to prevent skin abrasion, avoid the use of face masks for long periods if pre-existing dermatological conditions are present (37).

Our results are coherent with those of di Altobrando et al., as the preventive measures they proposed are directly in contrast with the risk factors emerged in our study, since adverse dermatological reactions to the mask were significantly higher in healthcare workers with a personal history of Acne or Seborrheic Dermatitis, and lower in those using a moisturizing face cream. We also explored the association between adverse dermatological effect to the mask, and masks reusage or applying disinfectant gel to the mask, but results were not statistically significant.

Daye et al. performed a cross-sectional study on healthcare workers during the COVID-19 pandemic, finding that skin problems were higher in professionals who did not use moisturizers; in their results was also highlighted that the use of PPE increased the severity of a pre-existing dermatological disease and the skin symptoms associated with them, and that skin problems were higher for healthcare workers who used PPEs, in female professionals and nurses (38).

In our study, similar findings emerged: using moisturizers is a protective factor against facial skin reactions to the masks, while these reactions appear to be higher in female healthcare workers and in those who had a pre-existing dermatological condition (i.e.,: Acne). We investigated a possible link between profession and dermatological reactions but our results were not statistically significant.

In our study a consistent result for both gloves and masks was that working in contact with Sars-CoV-2 patients increased the risk of a dermatological reaction. There are studies analyzing the PPE dermatological effect on healthcare professionals during the COVID-19 pandemic (37–39), but prevalence data on this phenomenon is scarce. Guertler et al. conducted a cross-sectional study comparing healthcare professionals working with COVID-19 patients and those who were not, reporting among results that there was no significant difference between these groups (40). In accordance with literature data, our study highlighted that working in contact with Sars-CoV-2 patients was a statistically significant risk factor (37–39); this might be caused by a longer use of PPEs in these workers, as well as by the use of more straining equipment, necessary to ensure the safety of HCWs working in contact with Sars-CoV-2 patients.

Prolonged use of PPEs has been reported to be a risk factor for dermatological reactions, and worsening of skin symptoms is directly correlated to the time PPEs are used (15, 21, 41). Coherently with these findings, we observed that adverse dermatological reactions to the hands were significantly correlated to wearing gloves between 3 and 6 h per day. The higher rate of dermatological reactions while wearing PPEs between 3 and 6 h might be because HCWs prepare for their shift and maintain their PPEs thorough their workday, and they might not have an appropriate timeframe to safely change their PPEs. An apparent lower risk of dermatological reactions to PPEs in HCWs above the 6 h mark might be due to the fact that these workers have time to change, and the physiologically needed breaks allow them to recover from the harm done by PPEs. Furthermore, workers wearing PPEs for more than 6 h a day might be better trained and informed concerning the proper and comfortable use of PPEs, thus avoiding relevant dermatological reactions. Further research is needed to further evaluate the correlation between PPEs time of usage and dermatological reactions.

The present study had some strengths and limitations. From an occupational health perspective, to our knowledge this was the first study to assess the eligibility of workers and the loss of working days due to dermatological illnesses, therefore contributing to assess the problem of occupational PPEs exposure during the COVID-19 pandemic. Furthermore, our sample was numerous (1,184 participants) and well-distributed concerning age groups, and working vs. not working with COVID-19 patients. On the other hand, gender distribution was not optimal, with females being far more represented in the sample than males; although the sample was numerous, geographical distribution could not be assessed.

## Conclusions

From this study's findings, the need to identify groups with a higher intrinsic risk of developing dermatologic reactions—such as nurses and female workers—during health surveillance visits has emerged, in order to ensure prevention measures are put in place for these workers to avoid the worsening of a pre-existing condition that might lead to morbidity and to the loss of working days. Furthermore, occupational physicians could identify workers subjected to other risk factors highlighted in this study, such as working with Sars-CoV-2 patients, prolonged use of PPEs, and pre-existing dermatological illnesses, in order to better prevent dermatological conditions in healthcare workers with a higher risk. This study can be a starting point for further research into occupational dermatology, to better comprehend which risk factors can be acted upon to prevent dermatologic reactions and loss of working days.

## Data Availability Statement

The raw data supporting the conclusions of this article will be made available by the authors, without undue reservation.

## Author Contributions

CP, IP, NS, and PS conceived and design the study and recollected data. IB performed the statistical analysis. MR, MG, CA, and AD drafted the paper. UM and WR supervised the study. All authors contributed to the article and approved the submitted version.

## Conflict of Interest

The authors declare that the research was conducted in the absence of any commercial or financial relationships that could be construed as a potential conflict of interest.

## Publisher's Note

All claims expressed in this article are solely those of the authors and do not necessarily represent those of their affiliated organizations, or those of the publisher, the editors and the reviewers. Any product that may be evaluated in this article, or claim that may be made by its manufacturer, is not guaranteed or endorsed by the publisher.
